# *Helicobacter pylori*-Induced Histone Modification, Associated Gene Expression in Gastric Epithelial Cells, and Its Implication in Pathogenesis

**DOI:** 10.1371/journal.pone.0009875

**Published:** 2010-04-01

**Authors:** Song-Ze Ding, Wolfgang Fischer, Maria Kaparakis-Liaskos, George Liechti, D. Scott Merrell, Patrick A. Grant, Richard L. Ferrero, Sheila E. Crowe, Rainer Haas, Masanori Hatakeyama, Joanna B. Goldberg

**Affiliations:** 1 Department of Microbiology, University of Virginia Health System, Charlottesville, Virginia, United States of America; 2 Max von Pettenkofer Institut, Munich, Germany; 3 Max von Pettenkofer Institute for Hygiene and Medical Microbiology, Ludwig-Maximilians-University, Munich, Germany; 4 Centre for Innate Immunity and Infectious Disease, Monash Institute of Medical Research, Clayton, Victoria, Australia; 5 Department of Microbiology and Immunology, Uniformed Service University of the Health Sciences, Bethesda, Maryland, United States of America; 6 Department of Biochemistry and Molecular Genetics, University of Virginia Health System, Charlottesville, Virginia, United States of America; 7 Divison of Gastroenterology and Hepatology, Department of Medicine, University of Virginia Health System, Charlottesville, Virginia, United States of America; 8 Division of Molecular Oncology, Institute for Genetic Medicine, Hokkaido University, Sapporo, Japan; University of Hyderabad, India

## Abstract

Histone modifications are critical in regulating gene expression, cell cycle, cell proliferation, and development. Relatively few studies have investigated whether *Helicobacter pylori*, the major cause of human gastric diseases, affects histone modification. We therefore investigated the effects of *H. pylori* infection on histone modifications in a global and promoter-specific manner in gastric epithelial cells. Infection of gastric epithelial cells by wild-type *H. pylori* induced time- and dose-dependent dephosphorylation of histone H3 at serine 10 (H3 Ser10) and decreased acetylation of H3 lysine 23, but had no effects on seven other specific modifications. Different *cag* pathogenicity island (PAI)-containing-clinical isolates showed similar abilities to induce H3 Ser10 dephosphorylation. Mutation of *cagA, vacA*, nonphosphorylateable CagA mutant *cagA_EPISA_*, or disruption of the flagella showed no effects, while deletion of the entire *cag*PAI restored the H3 Ser10 phosphorylation to control levels. Analysis of 27 *cag*PAI mutants indicated that the genes that caused H3 Ser10 dephosphorylation were similar to those that were previously found to induce interleukin-8, irrespective of CagA translocation. This effect was independent of ERK or p38 pathways and type I interferon signaling. Additionally, *c-Jun* and *hsp70* gene expression was associated with this histone modification. These results demonstrate that *H. pylori* alters histone modification and host response via a *cagA*-, *vacA*-independent, but *cag*PAI-dependent mechanisms, which contribute to its persistent infection and pathogenesis.

## Introduction

Chronic infection of the human stomach by *Helicobacter pylori*, a Gram-negative bacterium, is a major cause of chronic gastritis, peptic ulcers, and gastric malignancies, including gastric non-cardia adenocarcinoma and mucosal-associated lymphoid tissue (MALT) lymphoma [1]. *H. pylori* infection induces both acute and chronic gastritis, which is present as superficial mucosal inflammation in the gastric mucosa. *In vitro* experiments demonstrate that *H. pylori* activates multiple intracellular pathways including mitogen-activated protein kinases (MAPK), NF-κB and activator protein-1 as well as the Wnt/β-catenin pathway, which affect various cellular functions. These include increased inflammatory cytokine production, increased apoptosis, and epithelial cell turnover [2]. Bacterial virulence factors, such as cytotoxin-associated antigen (CagA), outer membrane proteins, the *cag* pathogenicity island (PAI), and vacuolating cytotoxin (VacA) are responsible for these effects [3]. *H. pylori* VacA has also been shown to inhibit T-cell proliferation and the cell cycle, and therefore suppress the immune response [4]. Upon infection, *H. pylori* induces the transcription of thousands of host genes, while at the same time represses another set of genes [5].

Immune subversion by histone modification is an important mechanism used by multiple bacteria and viruses during infection [6]. The accessibility to chromatin controls transcription factor-mediated gene expression and suppression, which is critical for normal cell function [7], [8]. For example, *Listeria monocytogenes* secretes listeriolysin O (LLO), which induces a dramatic dephosphorylation of histone H3 at serine 10 (H3 Ser10) and deacetylation of histone H4, and this correlates with changes in host gene expression during the early infection [9]. Other bacterial factors, including *Clostridium perfringens* toxin perfringolysin (PFO) and *Streptococcus pneumoniae* toxin pneumolysin (PLY) also induce the same dephosphorylation of histone H3 Ser10; this decreased phosphorylation of H3 Ser10 is associated with the previously reported decreased inflammatory cell responses during bacteria infection [9]. A recent report also indicates that *Shigella flexneri* toxin OspF blocks phosphorylation of MAPK ERK2 in the nucleus; this subsequently prevents histone H3 Ser10 phosphorylaton, which is a prerequisite of NF-κB activation and downstream gene transcription, and leads to a compromised inflammation in mouse tissue [10]. These results suggest a strategy commonly used by microbial pathogens to manipulate the host cellular function through histone modification and subversion of host innate immune responses for their survival or infection advantage.

One prominent feature of *H. pylori* infection is chronic and persistently enhanced inflammation with increased inflammatory cell infiltration in the local gastric mucosa and increased inflammatory cytokine production. A small percentage of infected individuals manifest the clinical presentation of gastritis, peptic ulcer, or gastric malignancy [2]. Recent studies have shown that histone remodeling by bacterial and viral pathogens is one mechanism of regulation of immune response during infection [8]. However whether *H. pylori* infection effects histone modifications has not been as thoroughly evaluated. In the present study, we investigated if *H. pylori* infection modulates host gastric epithelial cell histone modification. In addition, we correlated *H. pylori*-induced histone modification with the changes in gastric epithelial cell functions and detected bacterial genes that are responsible for these effects, and explore their impact on pathogenesis.

We demonstrate a *H. pylori cag*PAI-dependent dephosphorylation of histone H3 Ser10 and deacetylation of H3 lysine (K) 23. The effects of H3 Ser10 dephosphorylation are independent of major *H. pylori* virulence factors including CagA, VacA, and flagella. In addition, this *cag*PAI-dependent effect is common to multiple *H. pylori* strains, and the histone H3 Ser10 dephosphorylation is independent of ERK and p38 pathways, and type I interferon (IFN) signaling. Additionally, H3 Ser10 dephosphorylation is associated with changes in host gene expression. These results indicate a novel mechanism of *H. pylori* pathogenesis through histone modifications that has potential implications on *H. pylori-*induced chronic and persistent infections.

## Results

### *H. pylori cag*PAI-dependent induction of histone H3 Ser10 dephosphorylation in gastric epithelial cells

**Figure 1:**
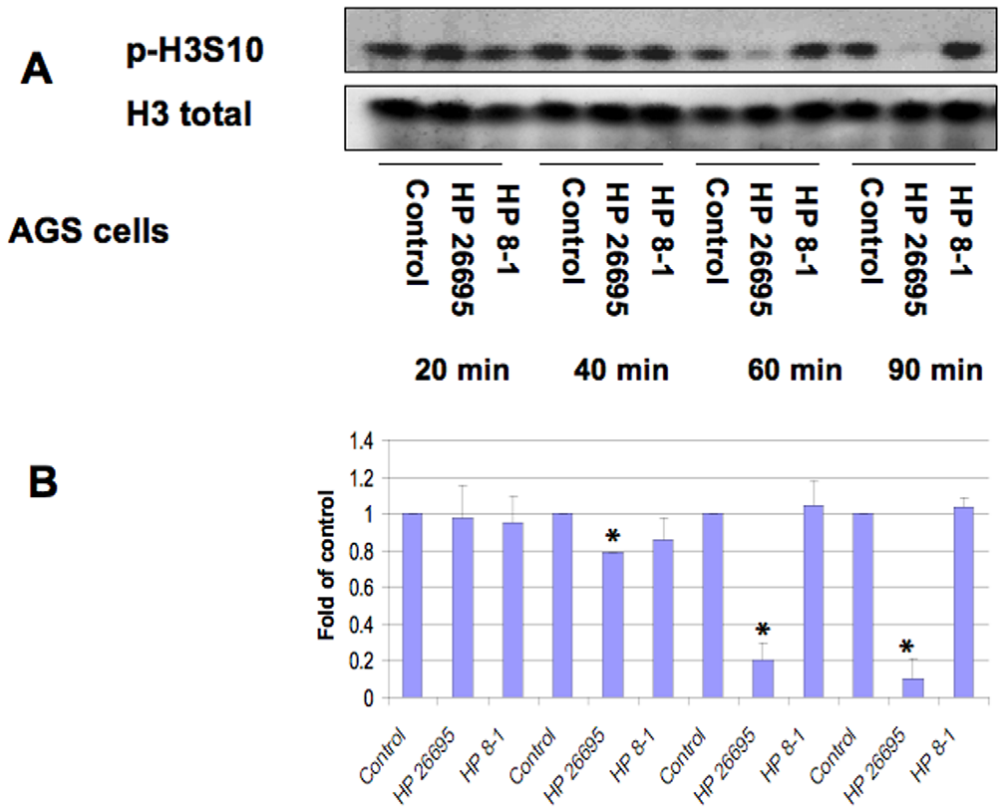
*H. pylori*-induced histone H3 Ser10 dephosphorylation is *cag*PAI-dependent in AGS cells. AGS cells (5×10^5^) were treated with medium alone, wild-type *H. pylori* 26695 (HP 26695), or an isogenic *cag* deletion strain (8-1) (HP 8-1) in antibiotic-free Ham's F-12 medium plus 5% FBS at MOI of 100:1 for various periods of time. The cells were washed, lysed and proteins were separated on a 15% SDS-polyacrylamide gel and transferred to nitrocellulose membrane, which was probed with rabbit anti-phospho-histone H3 Ser10 antibodies (p-H3S10). The original membrane was then stripped and re-probed with anti-total histone H3 antibodies to monitor protein loading. Blots are representative of three separate experiments with similar results (Panel A). Data are mean±SEM from three densitometry scans, adjusted with total histone H3, and expressed as fold changes over the appropriate control, *P<0.01 when compared with controls (Panel B).

**Figure 2:**
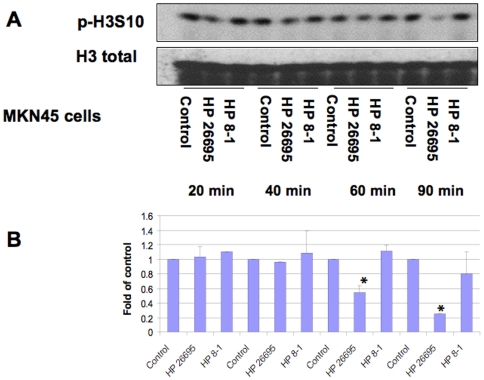
*H. pylori*-induced histone H3 Ser10 dephosphorylation is *cag*PAI-dependent in MKN45 cells. MKN45 cells (1×10^6^) were treated with medium alone, wild-type *H. pylori* 26695 (HP 26695), or an isogenic *cag* deletion strain (8-1) (HP 8-1) in RPMI-1640 medium plus 5% FBS at MOI of 100:1 for various periods of time. The cells were washed, lysed and proteins were separated on a 15% SDS-polyacrylamide gel and transferred to nitrocellulose membrane, which was probed with rabbit anti-phospho-histone H3 Ser10 antibodies (p-H3S10). The original membrane was then stripped and re-probed with anti-total histone H3 antibodies to monitor protein loading. Blots are representative of three separate experiments with similar results (Panel A). Data are mean±SEM from three densitometry scans, adjusted with total histone H3, and expressed as fold changes over the appropriate control, *P<0.01 when compared with controls (Panel B).

**Figure 3:**
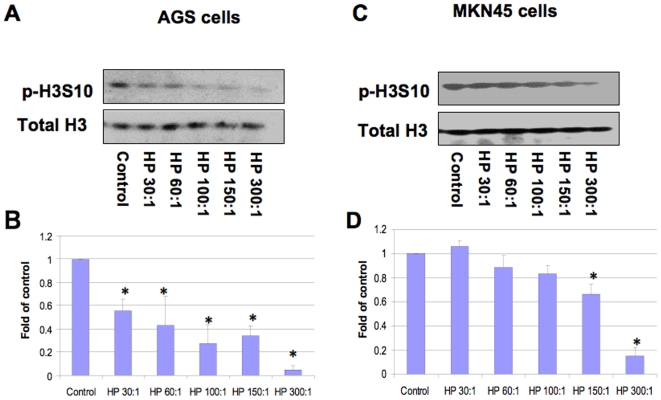
*H. pylori*-induced histone H3 Ser10 dephosphorylation is *cag*PAI-dependent in gastric epithelial cells. *H. pylori* 26695 was added at MOI of 30:1 to 300:1 MOI to AGS (5×10^5^) or MKN45 (1×10^6^) in antibiotic-free Hams F-12 or RPMI-1640 medium with 5% FBS, respectively, for 6 hours. The cells were washed, lysed and proteins were separated on a 15% SDS-polyacrylamide gel and transferred to nitrocellulose membrane, which was probed with rabbit anti-phospho-histone H3 Ser10 antibodies (p-H3S10). The original membrane was then stripped and re-probed with anti-total histone H3 antibodies to monitor protein loading. Blots are representative of three separate experiments with similar results (Panels A and C). Data are mean±SEM from three densitometry scans, adjusted with total histone H3, and expressed as fold changes over the appropriate control, *P<0.01 when compared with controls (Panels B and D).

In order to define the histone modifications induced by *H. pylori* in gastric epithelial cells, we first monitored the histone H3 Ser10 phosphorylation status in whole cell extracts by Western blot (Figures 1, 2, 3). AGS cells were infected with wild-type *H. pylori* 26695 or it's *cag*PAI deletion derivative 8-1, at an MOI of 100:1. The results showed that *H. pylori* 26695 time-dependently induced dephosphorylation of H3 Ser10 as early as 1 hour post-infection, and the effect was not seen in strain 8-1 (Figure 1). This effect was confirmed in MKN45 cells (Figure 2). In a dose-response study, we further confirmed *H. pylori* infection causes H3 Ser10 dephosphorylation in both AGS and MKN45 cells at an MOI of 30:1 to 300:1 (Figure 3). A significant difference was noted when the densitometry data were compared with control (*P<0.01). These results indicate that *H. pylori*-induces histone H3 Ser10 dephosphorylation and *cag*PAI is responsible for this effect.

### Effects of different *H. pylori* clinical isolates on H3 Ser10 dephosphorylation in AGS cells

**Figure 4:**
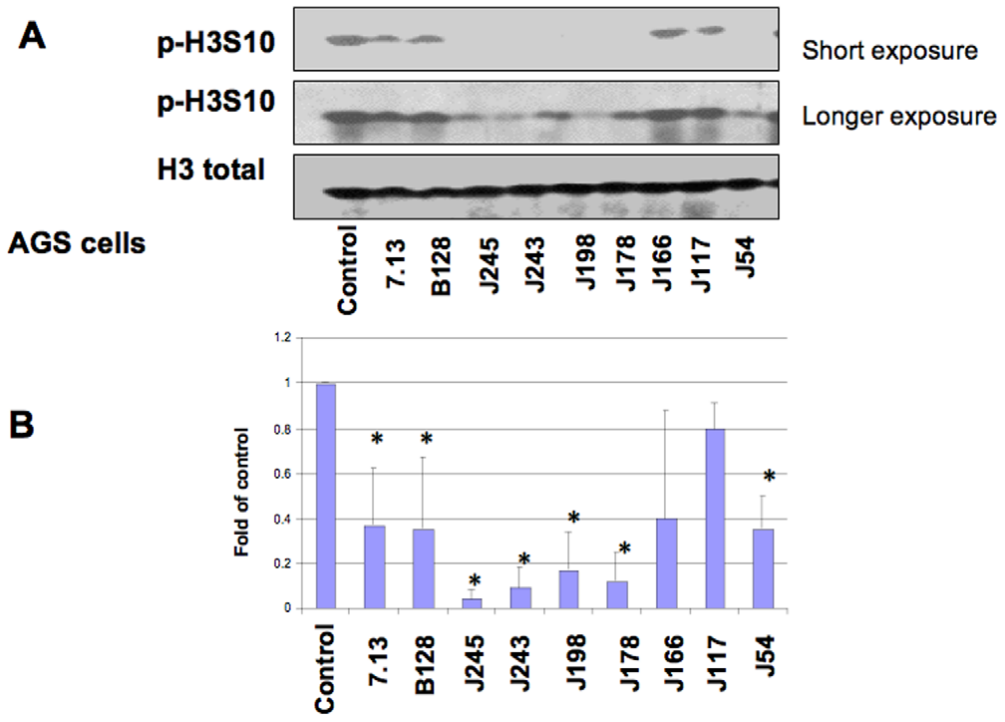
Effects of different *H. pylori* isolates on histone H3 Ser10 dephosphorylation in AGS cells. AGS cells (5×10^5^) were treated with medium alone or with 9 different *cag*+ clinical isolates at MOI of 150:1 for 6 hours. The cells were subsequently lysed and proteins were separated on 15% SDS-polyacrylamide gel, transferred to nitrocellulose membrane and probed with rabbit anti-phosphohistone H3 Ser10 (p-H3S10). Anti-total histone H3 antibodies (H3 total) were re-probed to monitor protein loading. Blots are representative of three separate experiments with similar results (Panel A). Data are mean±SEM from three densitometry scans, adjusted with total histone H3, and expressed as fold changes over the appropriate control, *P<0.01 when compared with controls (Panel B).

**Figure 5:**
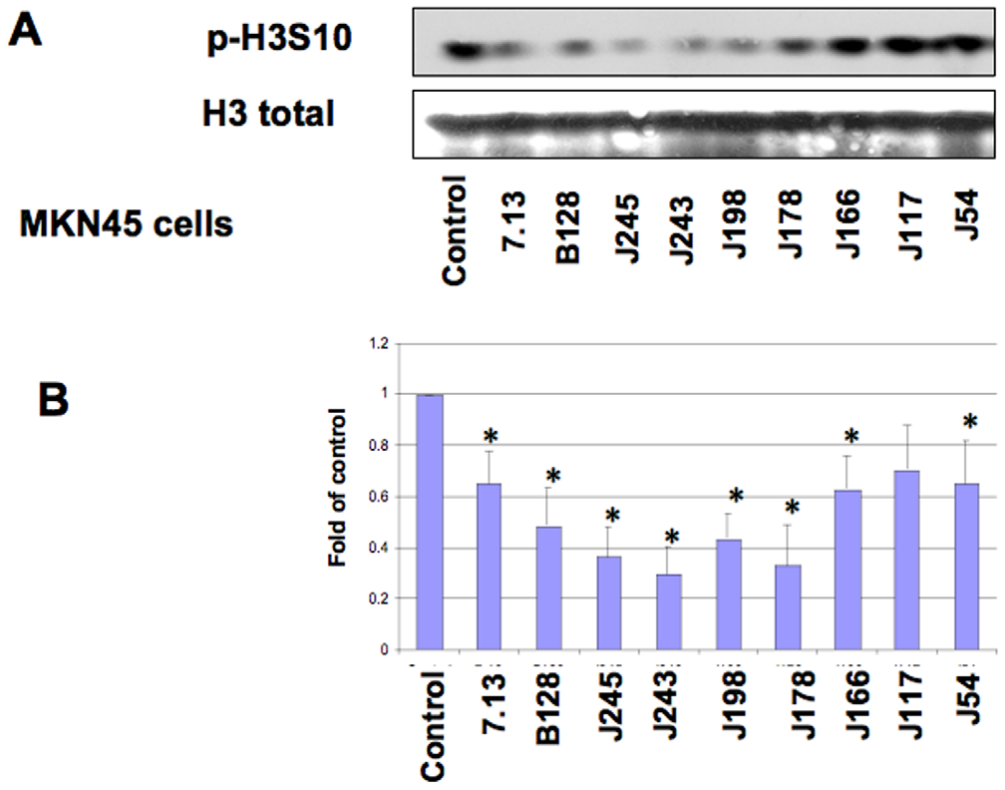
Effects of different *H. pylori* isolates on histone H3 Ser10 dephosphorylation in MKN45 cells. MKN45 cells (1×10^6^) were treated with medium alone or with 9 different *cag*+ clinical isolates at MOI of 150:1 for 6 hours. The cells were subsequently lysed and proteins were separated on 15% SDS-polyacrylamide gel, transferred to nitrocellulose membrane and probed with rabbit anti-phosphohistone H3 Ser10 (p-H3S10). Anti-total histone H3 antibodies (H3 total) were re-probed to monitor protein loading. Blots are representative of three separate experiments with similar results (Panel A). Data are mean±SEM from three densitometry scans, adjusted with total histone H3, and expressed as fold changes over the appropriate control, *P<0.01 when compared with controls (Panel B).

To further evaluate the potential strain-specific effects on *H. pylori*-induced H3 Ser10 dephosphorylation, we monitored the effects of different clinical isolates of *H. pylori* (Figures 4, 5). The results showed that seven out of nine different *H. pylori* clinical *cag*+ isolates induced consistent dephosphorylation of H3 Ser10 in both AGS (Figure 4) and MKN45 cells (Figure 5), albeit at various levels among different strains. A significant difference was noted when the densitometry data were compared with control cells for 7.13, B128, J245, J243, J198, J178, J166, and J54 strains in AGS and MKN45 cells (*P<0.01); the exception was strain J117 in both cell types. These results indicate that this effect is not limited to *H. pylori* strain 26695, but is also observed in multiple strains, suggesting that it is a property that is common among *H. pylori*.

### Effects of mutation in *H. pylori* virulence factors on H3 Ser10 dephosphorylation in AGS cells

**Figure 6:**
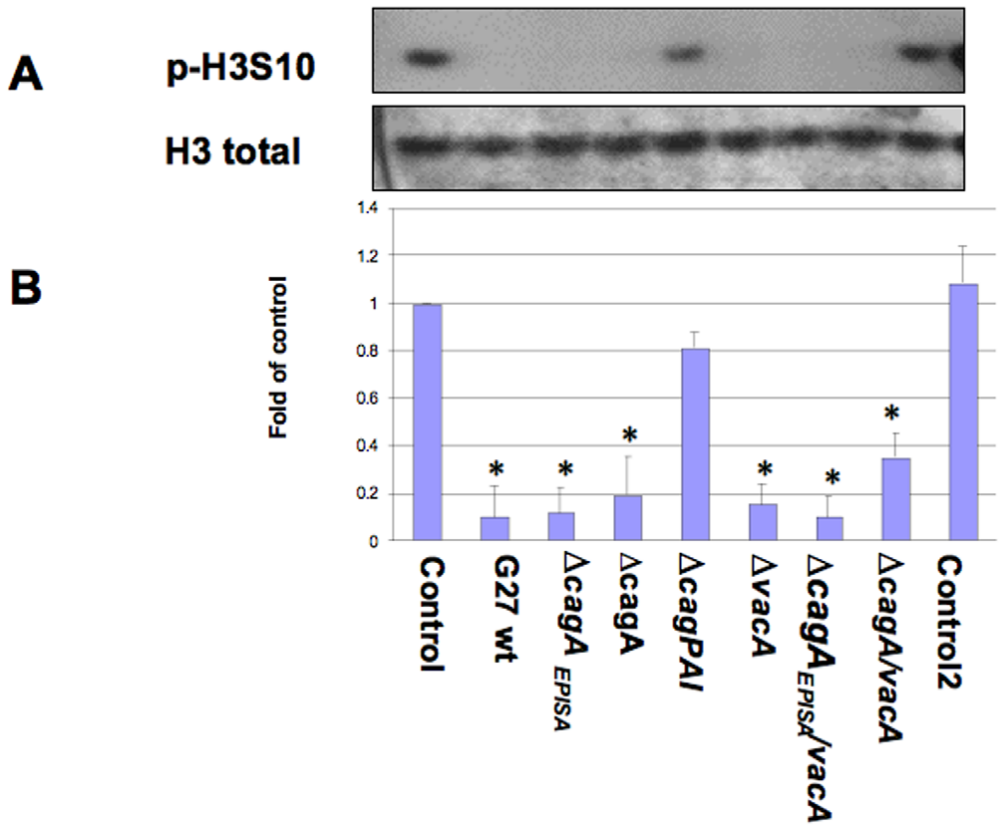
Effects of *H. pylori* mutants on histone H3 Ser10 dephosphorylation in AGS cells. AGS cells (5×10^5^) were treated with *H. pylori* G27-MA (G27) or its isogenic mutation strains in antibiotic-free medium without FBS for 6 hours at MOIs of 150:1. Control cells were treated with medium alone. The cells were washed, lysed and proteins were separated on a 15% SDS-polyacrylamide gel and transferred to nitrocellulose membrane, which was probed with rabbit anti-phospho-histone H3 Ser10 antibodies (p-H3S10). The original membrane was then stripped and re-probed with anti-total histone H3 antibodies to monitor protein loading. Photos are representative of two to four separate experiments with similar results (Panel A). Data are mean±SEM from densitometry scans, and expressed as fold changes over control without bacteria treatment, *P<0.01 when compared with controls (Panel B).

**Figure 7:**
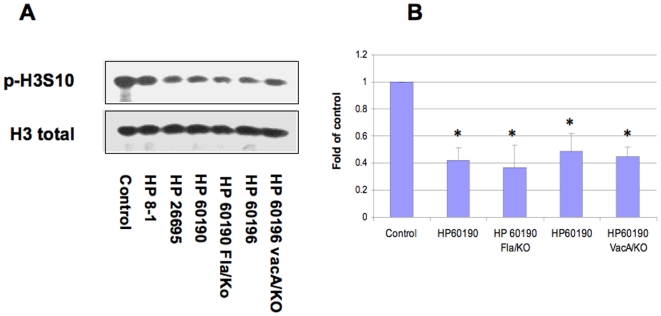
Effects of *H. pylori* mutations in *flaA* or *vacA* on histone H3 Ser10 dephosphorylation in AGS cells. AGS cells were treated with wild-type *H. pylori* strain 60190 (HP 60190) and its isogenic flagella mutant (HP 60190 FlaA/KO), 60190 and its isogenic *vacA* mutant (HP 60190 VacA/KO) for 6 hours. Control cells were treated with medium alone. The cells were washed, lysed and proteins were separated on a 15% SDS-polyacrylamide gel and transferred to nitrocellulose membrane, which was probed with rabbit anti-phospho-histone H3 Ser10 antibodies (p-H3S10). The original membrane was then stripped and re-probed with anti-total histone H3 antibodies to monitor protein loading. Photos are representative of two to four separate experiments with similar results (Panel A). Data are mean±SEM from densitometry scans, and expressed as fold changes over control without bacteria treatment, *P<0.01 when compared with controls (Panel B).

The major virulence factors of *H. pylori* including *cagA* and *vacA* have been shown to induce alterations in cellular signaling. To determine whether these factors are also responsible for the histone remodeling, we compared their ability to induce histone H3 Ser10 dephosphorylation by using Western blot. Wild-type strain G27-MA and its isogenic mutants in *cagA, vacA*, *cag*PAI, *cagA-vacA*, the nonphosphorylateable CagA mutant *cagA_EPISA_*, and *vacA-cagA_EPISA_* were assayed. The results showed that only mutation of *cag*PAI restored the H3 Ser10 phosphorylation status to approximately 80% of the uninfected control level, while *cagA, vacA*, *cagA-vacA*, the nonphosphorylateable CagA mutant *cagA_EPISA_*, and *vacA-cagA_EPISA_* still induced H3 Ser10 dephosphorylation (Figure 6). In addition, a 60190 mutant lacking flagella (due to a mutation of the *flaA* gene) did not alter the dephosphorylation of H3 Ser10 in AGS cells, compared to the wild-type 60190 strain (Figure 7). These results suggest that factors within the *cag*PAI, but independent of *cagA, vacA*, and flagella (*flaA*) are the cause of *H. pylori*-induced H3 Ser10 dephosphorylation.

Although we showed that *H. pylori* genes products within the *cag*PAI are the cause of H3 Ser10 dephosphorylation in AGS cells, G27-MA (and its derivatives) did not induce H3 Ser10 dephosphorylation in MKN45 cells (data not shown), likely due to the fact that G27-MA is a MDCK cell adherent strain [11], [12]. On the other hand, strain 26695 induced H3 Ser10 dephosphorylation in both cell types. In addition, *H. pylori* Sydney strain 1 which has a handicapped *cag*PAI [13], has no effect on histone dephosphorylation in AGS cells, while wild type P12 strain, which has a functional *cag*PAI, induced H3 Ser10 dephosphorylation (data not shown). The results suggest both pathogen and host factors may affect histone dephosphorylation.

### *H. pylori-*induced other histone modifications in gastric epithelial cells

**Figure 8:**
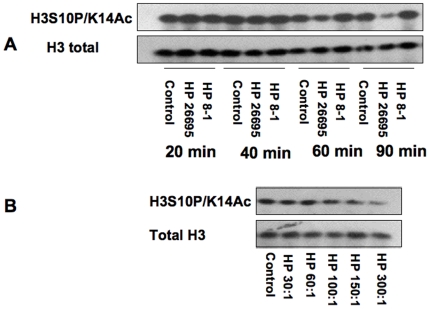
*H. pylori*-induced other histone modifications in AGS cells. AGS cells (5×10^5^) were treated with medium alone, wild-type *H. pylori* 26695 (HP 26695), an isogenic *cag* deletion strain 8-1 (HP 8-1) in antibiotic-free medium, plus 5% FBS at MOI of 100:1 (Panel A) or different MOIs (Panel B) for 6 hours. Cells were subsequently lysed and proteins were separated on 15% SDS-polyacrylamide gel and transferred to a nitrocellulose membrane. Various rabbit anti-histone modification antibodies were used for immunoblotting. Anti-total histone H3 antibodies were used to re-probe the membrane and monitor protein loading. Blots are representative of three separate experiments with similar results.

**Figure 9:**
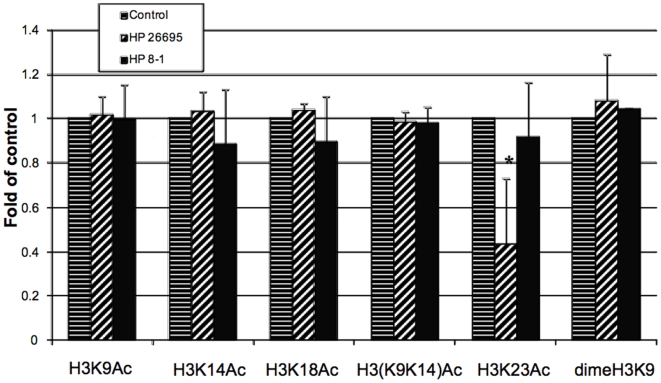
*H. pylori*-induced other histone modifications in gastric epithelial cells. AGS cells were treated as described in Figure 8 using various rabbit anti-histone modification antibodies. Data are mean±SEM from three assays, and expressed as fold changes over the appropriate control, *P<0.01 when compared with controls.

Noting that *H. pylori* can cause H3 Ser10 dephosphorylation, we next investigated if *H. pylori* 26695 also induced other histone modifications. We monitored several common histone protein modifications, including the acetylation of H3K9, H3K14, H3K18, H3K23, H3K9K14, H3S10P/K9Ac, H3S10P/K14Ac, dimethylation of H3K9, hyperacetylation of H4, and acetylation of H4K8 in gastric epithelial cells. The results indicated that *H. pylori* 26695 *cag*PAI induced dephosphorylation of H3S10P/K14Ac, in a similar manner to the H3 Ser10 dephosphorylation caused by *H. pylori* in both AGS (Figure 8) and MKN45 cells and that it was *cag*PAI-dependent (data not shown). We also detected decreased H3K23 acetylation during *H. pylori* infection in a *cag*PAI-dependent manner in AGS cells, while other modifications tested in this study revealed no difference as compared with uninfected control cells (Figure 9 and data not shown).

**Figure 10:**
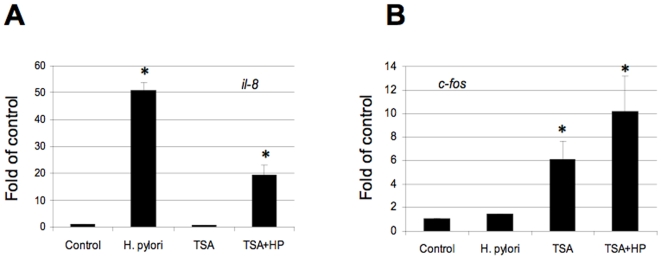
*H. pylori*-induced other histone modifications in gastric epithelial cells. MKN45 cells were stimulated with *H. pylori* 26695 or 8-1 as described in Figure 8 in the presence or absence of triscostatin A (TSA). Cells were collected for RNA extraction for subsequent quantitative RT-PCR assay using primers to *il-8* (Panel A) and *c-fos* (Panel B). Data are mean±SEM from three assays, and expressed as fold changes over the appropriate control, *P<0.01 when compared with controls.

Treatment of AGS or MKN45 cell with tricostatin A (TSA), a histone deacetylase inhibitor which non-specifically increases the chromatin acetylation status, resulted in increased multiple histone H3 acetylations (data not shown). Interestingly, this effect is associated with some commonly reported gene transcription pattern alterations in *il-8* and *c-fos* (Figure 10). We noted *il-8* gene expression was induced by *H. pylori* infection, while pre-treatment with TSA markedly reduced its level, suggesting the net effect of TSA treatment reduces *il-8* transcription. On the other hand, TSA increased *c-fos* transcription in the presence or absence of *H. pylori* infection. These results suggest that alteration of chromatin structure by increasing the acetylation status profoundly affect bacterial-induced host gene expression, and this effect is most likely a promoter-specific effect in gastric epithelial cells.

### *cag*PAI mutants and their effects on H3 Ser10 dephosphorylation in AGS cells

Since *cag*A is not responsible for the H3 Ser10 dephosphorylation in gastric epithelial cells as shown above, we investigated which gene or gene product within the *cag*PAI was responsible for this effect. To determine this, we used a series of *H. pylori cag* mutant strains [14] to test which gene(s) might cause the H3 Ser10 dephosphorylation. The results are presented in Table 1 and Figures S1 and S2. Among the 27 mutant strains tested, all *cag* genes that are necessary for IL-8 induction (522, 523, 525, 527, 528, 529, 530, 531, 532, 537, 539, 541, 544, 546) [14] were also required for complete H3 Ser10 dephosphorylation, only the 529, 537 and 544 genes do not seem to be completely essential, but these three have a rather low statistical significance (between 0.01 and 0.05). Likewise, all strains with mutations in genes that are not required for IL-8 induction (520, 521, 524, 526, 534, 535, 536, 540, 542, 543, 545 and 547) showed more or less complete H3 Ser10 dephosphorylation; only in the 520, 536, and 542 mutants was there not complete dephosphorylation. Mutants with reduced IL-8 induction (526, 538, 545) have also reduced H3 Ser10 dephosphorylation. The H3 dephosphorylation requirements are therefore similar to IL-8 induction requirements, irrespective of whether CagA can be translocated or not. These results clearly indicate the genes that might be responsible for causing this effect, and suggest there might be multiple processes involved, which are independent of CagA (Table 1 and Figures S1 and S2).

**Table 1:** Effects of *H. pylori cagPAI* mutants on histone H3 Ser10 dephosphorylation, CagA translocation and IL-8 induction in AGS cells.

Strain/mutant	H3 Ser10 dephosphorylation^a^	CagA translocation^b^	IL-8 induction^c^
26695 wt	++	++	++
Δ*cag*ξ (HP 520)	+	++	++
Δ*cag*ε (HP 521)	++	++	++
Δ*cag*δ (HP 522)	−	−	−
Δ*cag*γ (HP 523)	−	−	−
Δ*cag*β (HP 524)	++	−	++
Δ*cag*α (HP 525)	−	−	−
Δ*cagZ* (HP 526)	+	−	+
Δ*cagY* (HP 527)	−	−	−
Δ*cagX* (HP 528)	−	−	−
Δ*cagW* (HP 529)	(+)	−	−
Δ*cagV* (HP 530)	−	−	−
Δ*cagU* (HP 531)	−	−	−
Δ*cagT* (HP 532)	−	−	−
Δ*cagS* (HP 534)	++	++	++
Δ*cagQ* (HP 535)	++	++	++
Δ*cagP* (HP 536)	+	++	++
Δ*cagM* (HP 537)	(+)	−	−
Δ*cagN* (HP 538)	−	+	+
Δ*cagL* (HP 539)	−	−	−
Δ*cagI* (HP 540)	+	−	++
Δ*cagH* (HP 541)	−	−	−
Δ*cagG* (HP 542)	+	+	++
Δ*cagF* (HP 543)	++	−	++
Δ*cagE* (HP 544)	(+)	−	−
Δ*cagD* (HP 545)	(+)	−	+
Δ*cagC* (HP 546)	−	−	−
Δ*cagA* (HP 547)	++	−	++

AGS cells (5×10^5^) were treated in the presence or absence of wild-type *H. pylori* 26695 (26695) and its various isogenic mutants at an MOI of 150:1. The cell lysate was then subjected to immunoblot analysis with rabbit anti-phospho-histone H3 Ser10 antibodies. Anti-total histone H3 antibodies were used to re-probe the membrane and monitor protein loading. CagA translocation and the measurement of IL-8 by ELISA from culture supernatants were described in previous report [14]. The data are average result from 3-6 separate experiments.

a ++, >60% H3 Ser10 dephosphorylation; +, 60-40% H3 Ser10 dephosphorylation; -, <40% H3 Ser10 dephosphorylation (vs. non-infected); (+), 0.01<P<0.05.

b according to Fischer *et al.* [14]. CagA translocation; ++ efficient translocation, + significantly reduced translocation, - no translocation.

c IL-8 induction; ++ >60% induction, + 40–60% induction, −no or minimal induction (vs. wild-type).

### Gene expression associated with phospho-histone H3 Ser10 during *H. pylori* infection in AGS cells

**Figure 11:**
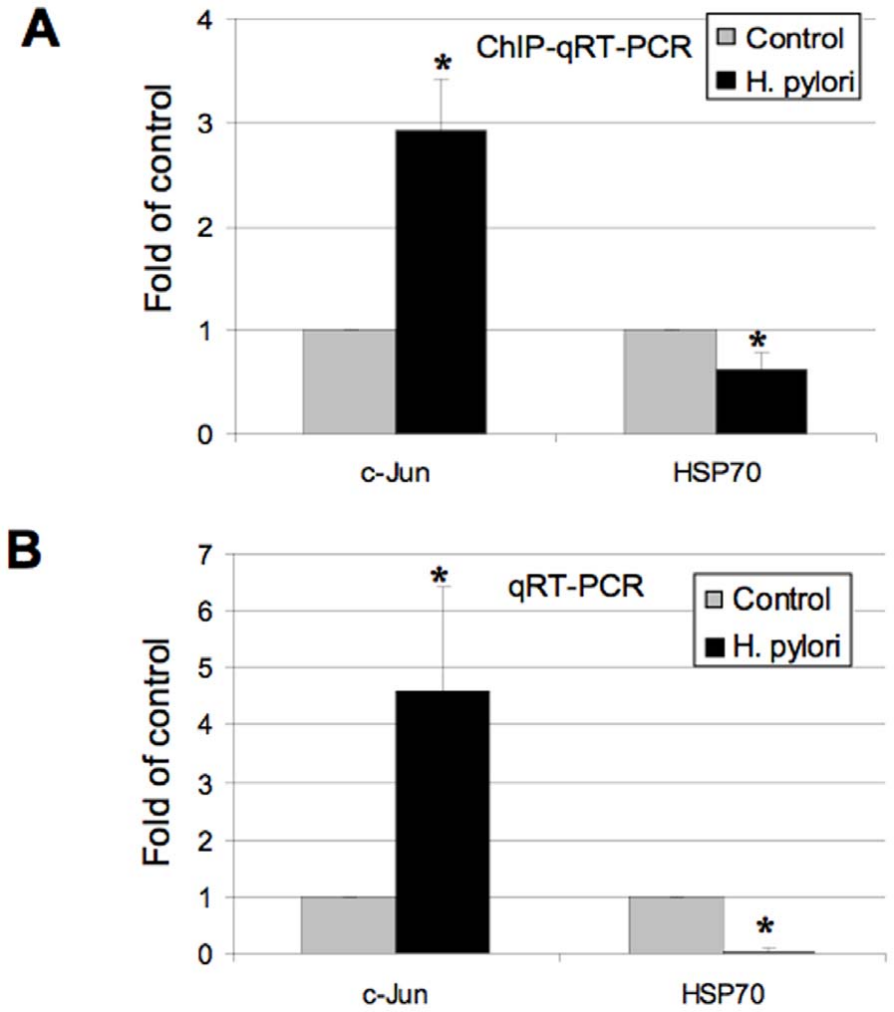
Gene expression associated with phospho-histone H3 Ser10 during *H. pylori* infection in AGS cells. AGS cells (5×10^6^) were treated with *H. pylori* G27-MA (G27) strains in antibiotic-free medium with 5% FBS at MOIs of 150:1. The cells are collected either for ChIP assay (Panel A) or quantitative RT-PCR analysis (Panel B) as described in Materials and Methods. For ChIP assay, cells were collected at 6 hours, and rabbit anti-phospho-histone H3 Ser10 (p-H3-Ser10) antibodies were used for immunoprecipitation (IP). Anti-total histone H3 or irrelevant IgG was used as monitoring control. Data represent the IP/total input ratio and expressed as fold changes over control without *H. pylori* treatment (Panel A). For quantitative RT-PCR, cells were collected at 6 hours RNA was extracted for PCR analysis. Data represent the relative mRNA expression, adjusted with HPRT and expressed as fold changes over control without *H. pylori* treatment (Panel B). Results are mean±SEM from 2-4 separate experiments, *P<0.01 when compared with controls.

To determine if the changes in histone modification are associated with alterations in host gene expression, we tested a group of genes that has previously reported to be changed during *H. pylori* or *L. monocytogenes* infection [9], [15] including, *cxcl2, prkdc, dusp4, cox2, c-Jun, hsp70, cyclin D1*, and *il-8*, using chromatin immunoprecipitation (ChIP). We infected AGS cells with wild-type *H. pylori* G27-MA, which showed dephosphorylation of H3 Ser 10, and performed RT-PCR and ChIP assays. Interestingly, we noted that *c-Jun* and *hsp70* gene expression was correlated with the H3 Ser10 phosphorylation in the promoter region of these genes (Figure 11). On the other hand, the gene expression pattern in histone H3 Ser10 phospho-antibody enriched material for *cxcl2, prkdc, dusp4, cox2, cyclin D1*, and *il-8* had no association with H3 Ser10 phosphorylation (data not shown). *c-Jun* and *hsp70* mRNA expression levels in *H. pylori*-stimulated AGS cells represented up- or down- regulated genes, respectively. The results are in line with previously reported gene array [5], [15] and the protein expression data [16], [17] and collectively suggests that *H. pylori* selectively regulates gene expression in host cells associated with chromatin modification, which subsequently regulates cellular functions.

### Effects of MAPK inhibition on phospho-H3 Ser10 expression during *H. pylori* infection in AGS cells

**Figure 12:**
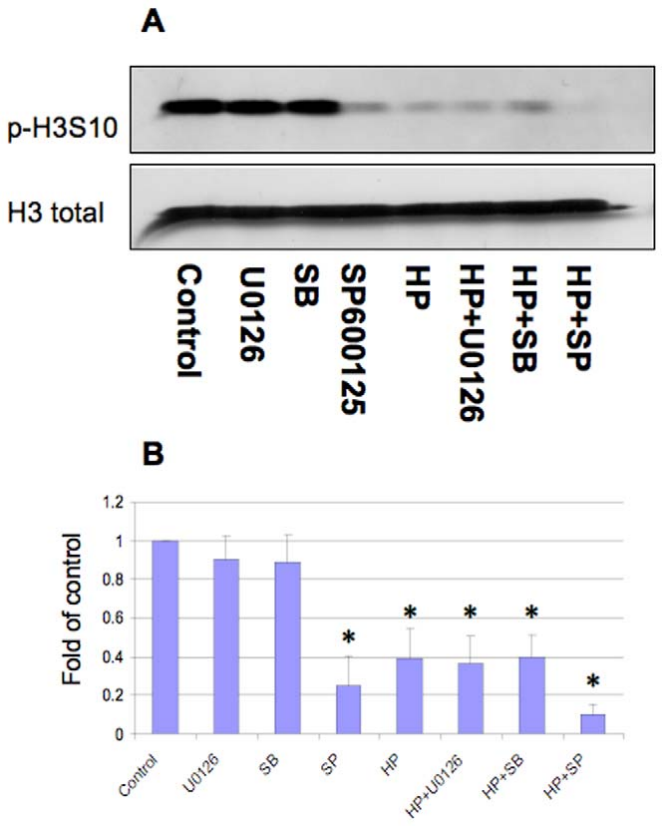
Effects of MAPK inhibition on phospho-histone H3 Ser10 expression during *H. pylori* infection in AGS cells. AGS cells (5×10^5^) were treated with *H. pylori* G27-MA (HP) strains in antibiotic-free medium with 5% FBS at MOIs of 100:1 for four hours. MAPK inhibitors including MEK1/2 inhibitor U0126 (5 µM), p38 inhibitor SB203580 (5 µM, SB) and JNK inhibitor SP600125 (25 µM, SP) were treated 30 minutes prior to bacteria stimulation. The cells were lysed and proteins were separated on 15% SDS-polyacrylamide gel for detection of phospho-histone H3 Ser10 (p-H3S10). Anti-total H3 antibodies were used to re-probe the membrane and monitor protein loading (H3 total). Blots are representative of three separate experiments with similar results (Panel A). Data are mean±SEM from densitometry scans, and expressed as fold changes over control without bacteria treatment (Panel B), *P<0.01 when compared with controls.

In order to identify the signaling pathways that mediate H3 Ser10 phosphorylation, we used ERK, p38, and JNK inhibitors to determine if these pathways might affect H3 Ser10 phosphorylation. The results indicate that only JNK inhibitor SP600125 blocked the H3 Ser10 phosphorylation regardless of the *H. pylori* infection status (Figure 12), suggesting that SP600125 or JNK pathway might be responsible for this event. ERK and p38 inhibition had no significant effects on H3 Ser 10 phosphorylation status on either basal or *H. pylori* stimulated cells.

### *H. pylori*-induced H3 Ser10 dephosphorylation is independent of IFN α/β signaling or bacterial DNA transfer into host cells

To further evaluate if *H. pylori* might use its type IV secretion system to deliver DNA into host cells [18] and trigger this histone modification, we evaluate the type I IFN (IFN α/β) signaling pathway activation upon bacterial stimulation, which is the hallmark of cellular response to foreign DNA stimulation [19]. The results suggest that no IFN signaling was induced, as both wild type *H. pylori* and its *cag*PAI mutant induced similar IFN α/β production by ELISA assay in mouse L929 cells 10 and 24 (data not shown) hours post-infection (Figures S3A and S3B). In addition, to avoid the cell type specific effects, we evaluated IFN α/β mRNA expression in the human gastric epithelial cell line AGS upon bacterial stimulation, and again, no obvious IFN α/β mRNA was induced by infection. On the other hand, the positive control, IL-8 mRNA, was strongly induced in the same set of samples, suggesting that *cag*PAI was functional during *H. pylori* infection (Figure S3C). These results suggest that *H. pylori* infection does not induce IFN α/β signaling or detectable bacterial DNA into host cell, even though it has a functional *cag*PAI and type IV secretion system.

## Discussion

Evidence has suggested the critical roles of histone remodeling in controlling diverse cellular functions. Histone modifications are necessary for the induction of host transcription programs, commonly known as the “histone code” [20], [21]. Phosphorylation of histone H3 and acetylation of lysines of histone H3 and H4 are frequently associated with gene transcription activation. Conversely, dephosphorylation and methylation of histones are more often linked with gene suppression [20], [21]. Targeting of histone remodeling and subsequent affects on the host cell immune response and limiting inflammation, has been shown to be a common strategy used by several bacteria, including *L. monocytogenes*, *C. perfringens*, *S. pneumoniae* and *S. flexneri* [9], [10]. Most of these bacteria secrete cytotoxins to induce the same dephosphorylation of histone H3 Ser10, while *S. flexneri* toxin blocks phosphorylation of histone H3 Ser10; these effects are associated with the mechanisms of altered host immune response [9], [10]. In this report, we add one more bacteria to this category, we note that *H. pylori cag*PAI is critical for this effect.

The *cag*PAI of *H. pylori* is a 40 kilobase region of the *H. pylori* genome and encodes about 30 genes including a type IV secretion system, which is important for its pathogenesis and is responsible for the transfer of CagA protein and peptidoglycan into host cell that induces subsequent intracellular signaling [13], [22]. CagA protein has profound effects on host cells, including its interaction with SHP-2 [23], activation of c-terminal Src kinase (CSK) [24], dephosphorylation of cortactin [25], and interaction with c-Met receptor or phospholipase C–γ [26], ZO-1 [11], and Grb-2 [27]. More recently, CagA has been shown to disrupt cell polarity through its binding to Par1/MarkII complex and also interacts with E-cadherin and deregulates the beta-catenin signaling that promotes intestinal transdifferentiation [28], [29]. However, it has also been noted that there is *cag*PAI-dependent but *cag*A-independent host signaling induced by *H. pylori*; of note is the activation of Rho-GTPase Rac1 and CDC 42, and activation of NF-κB by NOD1 signaling [13], [30]. Recently, *cag*PAI was also shown to mediate c-Met-induced gastric cancer cell invasiveness, independent of *cag*A during *H. pylori* infection in gastric epithelial cells [31]. The current results provide additional evidence of *cag*PAI-dependent yet *cagA*-, *vacA*-independent host responses.

Analysis of strains with mutations in the *cag*PAI allowed us to take a detailed look at the genes that might be responsible for the histone H3 Ser10 dephosphorylation. Among the 27 *cag*PAI mutants tested, most that caused histone H3 Ser10 dephosphorylation had been previously found to induce IL-8, however, this effect is not necessarily linked to CagA translocation (Table 2, and Figures S1 and S2). The IL-8 production during *H. pylori* infection has been shown to be mediated by peptidoglycan delivery through type IV secretion system *via* NOD1-mediated NF-κB activation [13]; this suggests that peptidoglycan might play a role in H3 Ser10 dephosphorylation. Since three mutants that cause H3 Ser10 dephosphorylation were neither related to CagA translocation nor IL-8 production, additional processes might be involved. This finding also suggests a complex interplay of *cag*PAI within the host cell. Future investigations focusing on this area are warranted to explore the underlying molecular and pathogenic mechanism.

**Table 2:** Primer sets used in this study.

Gene name	For RT-PCR (5′-3′)	For ChIP (5′-3′)
*hsp70*	fwd: CCAGCACGGAAAAGTCGAGA rev: GTGTTGGTGGGGTTCATTGC	fwd: GGAAGGTGCGGGAAGGTTCG rev: TTCTTGTCGGATGCTGGA
*c-jun*	fwd: CACGTTAACAGTGGGTGCCA rev: CCCCGACGGTCTCTCTTCA	fwd: AAAGCTATGTATGTATGTGCTGCAT rev: AACCGAGAGAACCTTCCTTTTTAT
*c-fos*	fwd: TCGGGCTTCAACGCAGACTA rev: GCAGTGACCGTGGGAATGAA	
*il-8*	fwd: GGCAGCCTTCCTGATTTCTG rev: GGGGTGGAAAGGTTTGGAGT	
*hIFN-*α	fwd: GCCTCGCCCTTTGCTTTACT rev: CTGTGGGTCTCAGGGAGATCA	
*hIFN-*β	fwd: ATGACCAACAAGTGTCTCCTCC rev: GCTCATGGAAAGAGCTGTAGTG	
Hprt*	fwd: TTGGAAAGGGTGTTTATTCCTCA rev: rev: TCCAGCAGGTCAGCAAAGAA	

Hprt*, hypoxanthine phosphoribosyltransferase.

Microbial pathogens modify host cellular processes to facilitate their own survival and to gain a proliferation advantage during the infection [6]. Often this is initiated by secreted toxins that mimic host proteins and functions, and usually target essential and well conserved molecules or signaling pathways. Dephosphorylation of H3 Ser10 is a general mechanism and it is correlated with suppressed host innate immune responses. However, the lack of inflammation at the site of infection is not the case of *H. pylori* infection, which is accompanied with the enhanced inflammation. Therefore, in the context of *H. pylori* infection, this dephosphorylation of histone H3 Ser10 may be linked with increased inflammatory response. This concept is supported by our ChIP data that shows it is correlated with *c-Jun* up-regulation and *hsp70* repression, whose functions are associated with increased inflammation. Nonetheless, whether this is a “fine tune” or “breaking effect”, or simply a bacteria strategy to enhance the inflammation process requires further clarification in the future.

The effects of specific histone modifications in host cells appears to be cell type and promoter specific: in the mouse macrophage, *H. pylori* peptidyl prolyl cis-, trans-isomerase (HP0175) has been shown to induce H3 Ser10 phosphorylation at the IL-6 promoter and this is also associated with increased IL-6 mRNA and protein expression [32]. *H. pylori* has been shown to regulate p21(WAF1) expression associated with histone H4 acetylation in gastric epithelial cells [33]. Previous reports also indicate that H3 Ser10 phosphorylation and H4 acetylation are associated with immediate-early gene, including *c-fos*, expression [34]. Additionally, intracellular *L. monocytogenes* has been shown to regulate *il-8* gene expression through histone modification, including increased H4K8 acetylation and phosphorylation of histone H3 Ser10 in endothelial cells [35]. In our current study, inhibiting histone deacetylase activity by using the histone deacetylase inhibitor TSA non-specifically increased multiple histone H3, H4 acetylations, and this was accompanied with altered gene transcription in both *il-8* and *c-fos* genes, including the up-regulation of *c-fos* and down-regulation of *il-8* in MKN45 cells upon *H. pylori* infection. These results suggest that altered chromatin structure can function to “mask” or “unmask” gene transcription start sites and therefore affects their transcription. The opposite effects between *il-8* and *c-fos* gene transcription provide additional information on the bacterial-induced gene regulation, and may suggest different roles of histone deacetylation/acetylation and transcription factors play on each of their gene promoter in gastric epithelial cells.

H3 Ser 10 is cell cycle mitotic marker in proliferating cells, and *H. pylori* has been shown to affect the cell cycle [17], [36], however, the relationship of H3 Ser 10 dephosphorylation and cell cycle arrest during *H. pylori* infection are not clear. Interestingly, during the submission of this work, a paper by Fehri *et al*. [37], indicated that *H. pylori*-induced *cag*PAI dependent H3 Ser10 dephosphorylation. H3 Ser10 dephosphorylation and cyclin B1 correlated with transient pre-mitotic arrest. Further they showed vaccinia-related kinase 1 (VRK1) activity but not Aurora B activity are responsible for this transient cell cycle delay. In addition, they pointed out that this effect was I*k*B kinase alpha (IKKα) dependent [37]. Since this effect is transient, whether this effect ultimately affect the cell cycle are not clear as several papers indicated that *H. pylori*-induced cell cycle arrest is independent of *cag*PAI [17], [36], and both VacA and CagA also affects the cell cycle progression. Although, this work focused on cell cycle, their other results are in line with the current work indicating that wild type *H. pylori*-induce a *cag*PAI dependent H3 Ser 10 dephosphorylation, and the current work further suggest this modification is also associated with gene transcription control. Since H3 Ser10 phosphorylation has been linked with both cell cycle and transcription control [7]–[10], [20], [21], we reason this could be a biphasic effect, which on the one hand impacts the cell cycle, on the other hand, influences transcription. However, how this biphasic effects contribute to the *H. pylori* pathogenesis remain to be established in the future.

MAPK pathways including ERK and p38 have been reported to mediate the increased H3 Ser 10 phosphorylation, but not decreased H3 Ser10 phosphorylation [9], [35]. Using MAPK inhibitors, which each block a specific signaling cascade [16], [17], we did not detect a significant effect of ERK and p38 inhibition on basal or *H. pylori*-induced H3 Ser10 phosphorylation levels. SP600125, the JNK inhibitor, which has been reported to reduce global H3 Ser10 phosphorylation [38], abolished the H3 Ser10 phorphorylation in the current study. The mechanism has yet to be identified as this effect by SP600125 has been reported to be very effective as early as 10 minute post-treatment and is independent of JNK activity [38]; this is probably indicative of either a JNK inhibition or pharmacological specific effect. Additionally, we noted *H. pylori-*induced a fast inhibition on H3 Ser10 phosphorylation within one hour, a time we reason most likely linked to transcriptional control through chromatin remodeling. Therefore, the current results provide a signaling link between bacterial infection and H3 Ser10 dephosphorylation.

The global gene expression profile regulated by phosphorylated histone H3 Ser10 has yet to be identified. In this work, we noted, several commonly expressed genes, including *il-8, cox2, cxcl2, prkdc, dusp4*, and *cyclin D1* were not associated with, or were independent of, histone modification in the current infection model. However, altered *c-jun* and *hsp70* gene expression was associated with the H3 Ser10 dephosphorylation. Increased c-Jun protein phosphorylation and mRNA and reduced *hsp70* mRNA has been observed during *H. pylori* infection [5], [15], [16]. c-Jun plays an important role during multiple cellular process, including inflammation, cell cycle, and proliferation. It forms a major AP-1 DNA binding protein and participates in the regulation of multiple inflammatory genes in gastric epithelial cells [16]. HSP 70 is a molecular chaperone that belongs to the heat shock protein family, and is important for the maintenance of cell integrity. Heat shock proteins are expressed in response to a range of cellular stresses [39]. Hsp70 exerts both anti-inflammatory and proinflammatory effects depending on the cell type, context, and cellular locations [39]. Its role in *H. pylori* infection is not clear, but several studies have suggested it has cytoprotective effects, by reducing stress-induced denaturation and aggregation of intracellular proteins, and protecting the mitochondria, and interfering with the stress-induced apoptotic program [40], [41]. It also protects against *H. pylori* infection by inhibiting the expression of iNOS from gastric epithelial cells [42]. The current study indicates its expression is associated with chromatin modifications and thus suggests another level of transcription control. Nonetheless, delineation of detailed functions requires further study. Chromatin immunoprecipitation array or promoter microarray may provide more information on the global gene expression profile that is associated with H3 Ser10 phosphorylation/dephosphorylation in gastric epithelial cells.

In conclusion, these observations provide novel evidence in microbial pathogenesis as well as *H. pylori-*induced pathophysiology in human stomach related to histone modifications. The delicate interplay between the host and pathogen may potentially lead to the different outcomes of *H. pylo*ri infection. Identification of the mechanisms by which *H. pylori* affects chromatin structure at gene promoters will allow us a better understanding of the gene transcription control, and subsequent alteration of cellular functions.

## Materials and Methods

### Cell lines, cell culture, and reagents

Tissue culture reagents were purchased from GIBCO (Invitrogen, Carlsbad, CA, USA). The human gastric epithelial cell line, AGS, and the mouse fibroblast cell line, L929, were purchased from American Type Culture Collection (ATCC, Manassas, VA, USA). The human gastric epithelial cell line MKN45 was purchased from JCRB Cell Bank (Osaka, Japan). Cells were grown in Ham's F-12 (AGS) or RPMI 1640 (MKN45 and L929) medium supplemented with 10% fetal bovine serum (FBS) without antibiotics at 37°C in a humidified 10% CO_2_ incubator.

MAPK inhibitors, including MEK1/2 inhibitor U0126, which inhibits extracellular signal-regulated kinases 1 and 2 (ERK1/2) activation, p38 inhibitor SB203580, and c-Jun/SAPK N-terminal kinases (JNK) inhibitor SP600125, were purchased from Calbiochem (La Jolla, CA, USA). Cells were treated with the above inhibitors 30 minutes before *H. pylori* stimulation.

Rabbit polyclonal anti-histone antibodies including histone H3 phosphorylated at serine 10 (p-H3 Ser10); histone H3 acetylated at lysine 9 (H3K9Ac); histone H3 acetylated at lysine 18 (H3K18Ac); histone H3 acetylated at lysine 23 (H3K23Ac); histone H3 phosphorylated at serine 10 and acetylated at lysine 9 (H3S10P/K9Ac); histone H3 phosphorylated serine 10 and acetylated lysine 14 (H3S10P/K14Ac); histone H3 dimethylated at lysine 9 (dimeH3K9); total H3; and histone H4 acetylated at lysine 8 (H4K8Ac) were purchased from Cell Signaling Technology (Beverly, MA, USA). Histone H3 acetylated at lysine 14 (H3K14Ac); histone H3 acetylated at lysine 9 and lysine 14 (H3(K9K14)Ac); and hyperacetylated H4 (H4Ac, penta) were purchased from Upstate Biotechnology (Charlottesville, VA, USA). Specific histone deacetylase inhibitor trichostatin A (TSA) was purchased from Sigma (St. Louis, MO, USA). Stock solutions were prepared in DMSO solution at 100 mM. In some experiments, cells were treated with the above inhibitors 30 minutes before *H. pylori* infection. Controls without inhibitors were treated with medium alone and an equal concentration of DMSO.

### *H. pylori* strains and infection

A total of 52 different *H. pylori* strains were used in the current study including 26695 and its isogenic *cag*PAI mutant (entire *cag* island deletion) strain 8-1 (kindly provided by Dr. Douglas Berg, Washington University School of Medicine, St. Louis, MO, USA) [43], wild-type *H. pylori* strain 60190 and its *vacA* mutant strain (60190 VacA/KO), which contains a kanamycin cassette insertion (kindly provided by Dr. Tim Cover, Vanderbilt University, Nashville, TN) [44], or flagella mutant strain 60190 FlaA/KO, as well as 9 clinical *cag*+ strains (7.13, B128, J245, J243, J198, J178, J166, J117, and J54) (kindly provided by Dr. Richard Peek Jr., Vanderbilt University) [45], strain G27-MA and isogenic strains with mutations in *cagA, vacA*, *cag*PAI, *cagA-vacA*, and the nonphosphorylateable CagA mutant *cagA_EPISA_* and *vacA-cagA_EPISA_* [11], [12], strain 26695 and its isogenic mutants in 27 *cag*PAI genes (Δ*cag*ξ, Δ*cag*ε, Δ*cag*δ, Δ*cag*γ, Δ*cag*β, Δ*cag*α, Δ*cagZ*, Δ*cagY*, Δ*cagX*, Δ*cagW*, Δ*cagV*, Δ*cagU*, Δ*cagT*, Δ*cagS*, Δ*cagQ*, Δ*cagP*, Δ*cagM*, Δ*cagN*, Δ*cagL*, Δ*cagI*, Δ*cagH*, Δ*cagG*, Δ*cagF*, Δ*cagE*, Δ*cagD*, Δ*cagC*, Δ*cagA*), and strain P12, its Δ*cagA* and Δ*cagPAI* mutants [14], [46]. All bacteria were grown for 2 days on sheep blood agar plates (Remel Inc., Lenexa, KS, USA), in 10% CO_2_ at 37°C, and then harvested with a sterile cotton swab and resuspended in phosphate buffered-saline (PBS) solution. The bacteria were pelleted at 1,400×*g* for 10 minutes and resuspended in 5 ml of culture medium and added to the cell culture media at different bacteria to cell ratios (multiplicity of infection, MOI), as indicated. Infections lasted from 0.5 to 6 hours in 10% CO_2_ at 37°C. Under these conditions, *H. pylori* remained alive and motile (with the exception of the flagella mutant, which, as expected, was nonmotile). We also noted that wild-type 26695, P12, and G27-MA strains induced hummingbird phenotype.

### Western blot analysis of histone protein modification

AGS cells (5×10^5^) or MKN45 cells (1×10^6^) per well in 6-well culture plates were infected with bacteria at designated doses for designated times, washed three times with PBS, and lysed with cell lysis buffer directly on the dish (62.5 mM Tris-HCl (pH 6.8), 2% SDS, 10% glycerol, 50 mM dithiothreitol, 0.1% bromophenol blue). Prior to loading, samples were boiled at 100°C for 3 minutes, cooled down on ice, and then separated on 15% SDS polyacrylamide gels. Proteins were subsequently transferred from gels onto nitrocellulose membranes (Bio-Rad). Membranes were blocked for 1 hour at room temperature in Tris-buffered saline plus 0.025% Tween-20 (TBS-T) with 5% nonfat dry milk (pH 7.4). Various histone antibodies were diluted at 1∶1000 in TBS-T with 5% nonfat dry milk solution. Membranes were then incubated with antibodies at 4°C overnight and washed three times with TBS-T (pH 7.4). The secondary antibodies, horseradish peroxidase (HRP)-conjugated goat anti-rabbit antibodies, were used at a 1∶1000 dilution in TBS-T with 5% nonfat dry milk and incubated at room temperature for 3 hours (pH 7.4). Bands were detected with an enhanced chemiluminescence detection kit (Perkin Elmer Life Sciences, Boston, MA). In some experiments, proteins were separated on 8% polyacrylamide gel, blotted on a polyvinylidene difluoride membrane, and examined for p-H3 Ser10 with protein A coupled to alkaline phosphatase. The original membrane was stripped with stripping buffer (50 mM Tris-HCl (pH 6.7), 2% SDS, and 200 mM β-mercaptoethanol) at 60°C for 30 minutes, followed by three washes with TBS-T and blocked for 1 hour with TBS-T in 5% nonfat dry milk solution (pH 7.4) and re-probed with total histone H3 antibodies to assess protein loading.

### Quantitative RT-PCR for gene expression

Total RNA from AGS and MKN45 cells was purified using the RNeasy Mini Kit (Qiagen), as described previously [15], [47]. Reverse transcription of 0.5 µg of total cellular RNA was performed in a final volume of 20 µl containing 5x first strand buffer (Invitrogen), 1 mM of each dNTP, 20 units of placental RNase inhibitor, 5 µM random hexamer, and 9 units of Moloney murine leukemia virus reverse transcriptase (Invitrogen). After incubation at 37°C for 45 minutes, the samples were heated for 5 minutes at 92°C to end the reaction, diluted at 1∶4 and stored at −20°C until PCR use. Two µl of cDNA was subjected to real-time quantitative PCR using the Opticon system (MJ Research, Waltham, MA, USA) with SYBR Green I (Molecular Probes, Eugene, OR, USA) as a fluorescent reporter. Threshold cycle number of duplicate reactions was determined using the Opticon software and levels of selected gene mRNA expression were normalized to hypoxanthine phsophoribosyltransferase (HPRT) levels using the formula 2(*Rt*–*Et*), where *Rt* is the mean threshold cycle for the reference gene HPRT and *Et* is the mean threshold cycle for the experimental gene. Data were expressed as arbitrary units and fold changes were adjusted to the non-stimulated control cells. Primer sequences are provided in Table 2.

### Enzyme-linked immunosorbent assay (ELISA)

Cells were seeded and treated essentially the same way as described for Western blot. The procedures to detect IL-8 production from AGS cell was described previously [14]; for mouse IFN α/β measurement from L929 cells, we followed the manufacturer's protocol (PBL InterferonSource, Piscataway, NJ, USA).

### Chromatin immunoprecipitation (ChIP) assay

Chromatin immunoprecipitation (ChIP) assays were performed as previously described [9], [48]. Briefly, AGS cells (5×10^6^) per well in 150 mm dish were infected with bacteria at MOI of 150:1 for 6 hours, washed three times with PBS, and cross-linked with 1% formaldehyde (Sigma) for 10 minutes. Cells were harvested in cell lysis buffer, and sonicated with 6×10 second pulses to generate 0.4–1.2 kb size. The sonicated chromatin was then split equally into four parts, one part without any process served as input control, the other 3 parts were immunoprecipitated with: rabbit polyclonal histone H3 Ser10 antibody (Abcam, Cambridge, MA, USA #ab12191) [9], rabbit polyclonal total H3 antibody (#ab1791, Abcam), and non-immune isogenic IgG, at 4°C for 12 hours. DNA/protein complexes were captured by Dynabeads Protein A (Invitrogen). The samples were then washed, eluted in SDS Elution Buffer, and the cross-links reversed by overnight incubation at 65°C. DNA was purified using phenol:chloroform extraction and ethanol precipitation. Quantitative real-time RT-PCR was performed from input and ChIP material. DNA content in immunoprecipitation (IP) samples was measured relative to the total input; the data were expressed as fold changes of ratio of IP/input in each condition and adjusted with non-*H. pylori* treated control. Total H3 and non-immune isogenic IgG was used as monitoring control. PCR primer for both ChIP and RT-PCR are listed in Table 2.

### Statistical analysis

All quantitative data were expressed as mean ± SEM. Data were compared by using paired or unpaired Student *“t”* tests, and differences were considered significant if *P* values were <0.05.

## Supporting Information

Figure S1 Effects of *cag*PAI mutants on H3 Ser 10 dephosphorylation during *H. pylori* infection in AGS cells. AGS cells (5×10^5^) were treated in the presence or absence of wild-type *H. pylori* 26695 and its various mutants strains at MOI of 150:1. The cell lysate was then subjected to immunoblot analysis with rabbit anti-phospho-histone H3 Ser10 antibodies, anti-total H3 antibodies were used to re-probe the membrane and monitor protein loading. A representative blot from each strain is presented.
(0.18 MB TIF)

Figure S2 Comparison of the effects of *cag*PAI mutants on H3 Ser 10 dephosphorylation during *H. pylori* infection in AGS cells. Data are mean±SEM from 3–6 densitometry scans, adjusted with total histone H3, and expressed as fold changes over the appropriate control. **P<0.05, *P<0.01 when compared with controls.
(0.25 MB TIF)

Figure S3 *H. pylori*-induced H3 Ser10 dephosphorylation is independent of IFN α/β signaling or bacterial DNA transfer into host cells. Mouse fibroblast cell line L929 (2×10^5^) and human gastric epithelial cell line AGS (5×10^5^) were treated with *H. pylori* G27-MA (G27) or its isogenic mutant strains in antibiotic-free medium for 10 hours at an MOI of 100:1, control cells were treated with medium alone. Supernatant from L929 cells were collected and used to measure IFN α/β production (panels A and B). RNA was extracted from AGS cells with the same treatment and cDNA was made for quantitative RT-PCR assay as described in Materials and Methods. Data are mean±SEM from two duplicate determinants, and PCR data are expressed as fold changes over control without bacteria treatment (panel C). *P<0.01 when compared with controls.
(0.21 MB TIF)

## References

[1] Peek RM, Crabtree JE (2006). *Helicobacter* infection and gastric neoplasia.. J Pathol.

[2] Ernst PB, Peura DA, Crowe SE (2006). The translation of *Helicobacter pylori* basic research to patient care.. Gastroenterology.

[3] Lu H, Yamaoka Y, Graham DY (2005). *Helicobacter pylori* virulence factors: Facts and fantasies.. Curr Opin Gastroenterol.

[4] Gebert B, Fischer W, Weiss E, Hoffmann R, Haas R (2003). *Helicobacter pylori* vacuolating cytotoxin inhibits T lymphocyte activation.. Science.

[5] Guillemin K, Salama NR, Tompkins LS, Falkow S (2002). Cag pathogenicity island-specific responses of gastric epithelial cells to *Helicobacter pylori* infection.. Proc Natl Acad Sci U S A.

[6] Bhavsar AP, Guttman JA, Finlay BB (2007). Manipulation of host-cell pathways by bacterial pathogens.. Nature.

[7] Mizzen CA, Allis CD (2000). Transcription. new insights into an old modification.. Science.

[8] Sansonetti PJ, Di Santo JP (2007). Debugging how bacteria manipulate the immune response.. Immunity.

[9] Hamon MA, Batsche E, Regnault B, Tham TN, Seveau S (2007). Histone modifications induced by a family of bacterial toxins.. Proc Natl Acad Sci U S A.

[10] Arbibe L, Kim DW, Batsche E, Pedron T, Mateescu B (2007). An injected bacterial effector targets chromatin access for transcription factor NF-kappaB to alter transcription of host genes involved in immune responses.. Nat Immunol.

[11] Amieva MR, Vogelmann R, Covacci A, Tompkins LS, Nelson WJ (2003). Disruption of the epithelial apical-junctional complex by *Helicobacter pylori* CagA.. Science.

[12] El-Etr SH, Mueller A, Tompkins LS, Falkow S, Merrell DS (2004). Phosphorylation-independent effects of CagA during interaction between *Helicobacter pylori* and T84 polarized monolayers.. J Infect Dis.

[13] Viala J, Chaput C, Boneca IG, Cardona A, Girardin SE (2004). Nod1 responds to peptidoglycan delivered by the *Helicobacter pylori* cag pathogenicity island.. Nat Immunol.

[14] Fischer W, Puls J, Buhrdorf R, Gebert B, Odenbreit S (2001). Systematic mutagenesis of the *Helicobacter pylori* cag pathogenicity island: Essential genes for CagA translocation in host cells and induction of interleukin-8.. Mol Microbiol.

[15] Ding SZ, Torok AM, Smith MF, Goldberg JB (2005). Toll-like receptor 2-mediated gene expression in epithelial cells during *Helicobacter pylori* infection.. Helicobacter.

[16] Ding SZ, Olekhnovich IN, Cover TL, Peek RM, Smith MF (2008). *Helicobacter pylori* and mitogen-activated protein kinases mediate activator protein-1 (AP-1) subcomponent protein expression and DNA-binding activity in gastric epithelial cells.. FEMS Immunol Med Microbiol.

[17] Ding SZ, Smith MF, Goldberg JB (2008). *Helicobacter pylori* and mitogen-activated protein kinases regulate the cell cycle, proliferation and apoptosis in gastric epithelial cells.. J Gastroenterol Hepatol.

[18] Covacci A, Rappuoli R (2000). Tyrosine-phosphorylated bacterial proteins: Trojan horses for the host cell.. J Exp Med.

[19] Takaoka A, Wang Z, Choi MK, Yanai H, Negishi H (2007). DAI (DLM-1/ZBP1) is a cytosolic DNA sensor and an activator of innate immune response.. Nature.

[20] Fischle W, Wang Y, Allis CD (2003). Histone and chromatin cross-talk.. Curr Opin Cell Biol.

[21] Jenuwein T, Allis CD (2001). Translating the histone code.. Science.

[22] Segal ED, Cha J, Lo J, Falkow S, Tompkins LS (1999). Altered states: Involvement of phosphorylated CagA in the induction of host cellular growth changes by *Helicobacter pylori*.. Proc Natl Acad Sci U S A.

[23] Higashi H, Tsutsumi R, Muto S, Sugiyama T, Azuma T (2002). SHP-2 tyrosine phosphatase as an intracellular target of *Helicobacter pylori* CagA protein.. Science.

[24] Tsutsumi R, Higashi H, Higuchi M, Okada M, Hatakeyama M (2003). Attenuation of *Helicobacter pylori* CagA x SHP-2 signaling by interaction between CagA and C-terminal src kinase.. J Biol Chem.

[25] Selbach M, Moese S, Hurwitz R, Hauck CR, Meyer TF (2003). The *Helicobacter pylori* CagA protein induces cortactin dephosphorylation and actin rearrangement by c-src inactivation.. EMBO J.

[26] Churin Y, Al-Ghoul L, Kepp O, Meyer TF, Birchmeier W (2003). *Helicobacter pylori* CagA protein targets the c-met receptor and enhances the motogenic response.. J Cell Biol.

[27] Mimuro H, Suzuki T, Tanaka J, Asahi M, Haas R (2002). Grb2 is a key mediator of *Helicobacter pylori* CagA protein activities.. Mol Cell.

[28] Murata-Kamiya N, Kurashima Y, Teishikata Y, Yamahashi Y, Saito Y (2007). *Helicobacter pylori* CagA interacts with E-cadherin and deregulates the beta-catenin signal that promotes intestinal transdifferentiation in gastric epithelial cells.. Oncogene.

[29] Saadat I, Higashi H, Obuse C, Umeda M, Murata-Kamiya N (2007). *Helicobacter pylori* CagA targets PAR1/MARK kinase to disrupt epithelial cell polarity.. Nature.

[30] Churin Y, Kardalinou E, Meyer TF, Naumann M (2001). Pathogenicity island-dependent activation of rho GTPases Rac1 and Cdc42 in *Helicobacter pylori* infection.. Mol Microbiol.

[31] Oliveira MJ, Costa AC, Costa AM, Henriques L, Suriano G (2006). *Helicobacter pylori* induces gastric epithelial cell invasion in a c-met and type IV secretion system-dependent manner.. J Biol Chem.

[32] Pathak SK, Basu S, Bhattacharyya A, Pathak S, Banerjee A (2006). TLR4-dependent NF-kappaB activation and mitogen- and stress-activated protein kinase 1-triggered phosphorylation events are central to *Helicobacter pylori* peptidyl prolyl cis-, trans-isomerase (HP0175)-mediated induction of IL-6 release from macrophages.. J Immunol.

[33] Xia G, Schneider-Stock R, Diestel A, Habold C, Krueger S (2008). *Helicobacter pylori* regulates p21(WAF1) by histone H4 acetylation.. Biochem Biophys Res Commun.

[34] Thomson S, Clayton AL, Mahadevan LC (2001). Independent dynamic regulation of histone phosphorylation and acetylation during immediate-early gene induction.. Mol Cell.

[35] Schmeck B, Beermann W, van Laak V, Zahlten J, Opitz B (2005). Intracellular bacteria differentially regulated endothelial cytokine release by MAPK-dependent histone modification.. J Immunol.

[36] Ahmed A, Smoot D, Littleton G, Tackey R, Walters CS (2000). *Helicobacter pylori* inhibits gastric cell cycle progression.. Microbes Infect.

[37] Fehri LF, Rechner C, Janssen S, Mak TN, Holland C (2009). *Helicobacter pylori*-induced modification of the histone H3 phosphorylation status in gastric epithelial cells reflects its impact on cell cycle regulation.. Epigenetics.

[38] Huang W, Batra S, Korrapati S, Mishra V, Mehta KD (2006). Selective repression of low-density lipoprotein receptor expression by SP600125: Coupling of histone H3-Ser10 phosphorylation and Sp1 occupancy.. Mol Cell Biol.

[39] Giffard RG, Han RQ, Emery JF, Duan M, Pittet JF (2008). Regulation of apoptotic and inflammatory cell signaling in cerebral ischemia: The complex roles of heat shock protein 70.. Anesthesiology.

[40] Pierzchalski P, Krawiec A, Ptak-Belowska A, Baranska A, Konturek SJ (2006). The mechanism of heat-shock protein 70 gene expression abolition in gastric epithelium caused by *Helicobacter pylori* infection.. Helicobacter.

[41] Rokutan K (2000). Role of heat shock proteins in gastric mucosal protection.. J Gastroenterol Hepatol.

[42] Yeo M, Park HK, Kim DK, Cho SW, Kim YS (2004). Restoration of heat shock protein70 suppresses gastric mucosal inducible nitric oxide synthase expression induced by *Helicobacter pylori*.. Proteomics.

[43] Akopyants NS, Clifton SW, Kersulyte D, Crabtree JE, Youree BE (1998). Analyses of the cag pathogenicity island of *Helicobacter pylori*.. Mol Microbiol.

[44] Cover TL, Tummuru MK, Cao P, Thompson SA, Blaser MJ (1994). Divergence of genetic sequences for the vacuolating cytotoxin among *Helicobacter pylori* strains.. J Biol Chem.

[45] Franco AT, Israel DA, Washington MK, Krishna U, Fox JG (2005). Activation of beta-catenin by carcinogenic *Helicobacter pylori*.. Proc Natl Acad Sci U S A.

[46] Odenbreit S, Gebert B, Puls J, Fischer W, Haas R (2001). Interaction of *Helicobacter pylori* with professional phagocytes: Role of the cag pathogenicity island and translocation, phosphorylation and processing of CagA.. Cell Microbiol.

[47] Smith MF, Mitchell A, Li G, Ding S, Fitzmaurice AM (2003). Toll-like receptor (TLR) 2 and TLR5, but not TLR4, are required for *Helicobacter pylori*-induced NF-kappa B activation and chemokine expression by epithelial cells.. J Biol Chem.

[48] To KK, Polgar O, Huff LM, Morisaki K, Bates SE (2008). Histone modifications at the ABCG2 promoter following treatment with histone deacetylase inhibitor mirror those in multidrug-resistant cells.. Mol Cancer Res.

