# Supplementary figures and images for: Helicobacter pylori-Induced Histone Modification, Associated Gene Expression in Gastric Epithelial Cells, and Its Implication in Pathogenesis
Source: PLoS One. 2010 Apr 1;5(4):e9875. doi: 10.1371/journal.pone.0009875 (PMC2848570; doi:10.1371/journal.pone.0009875)

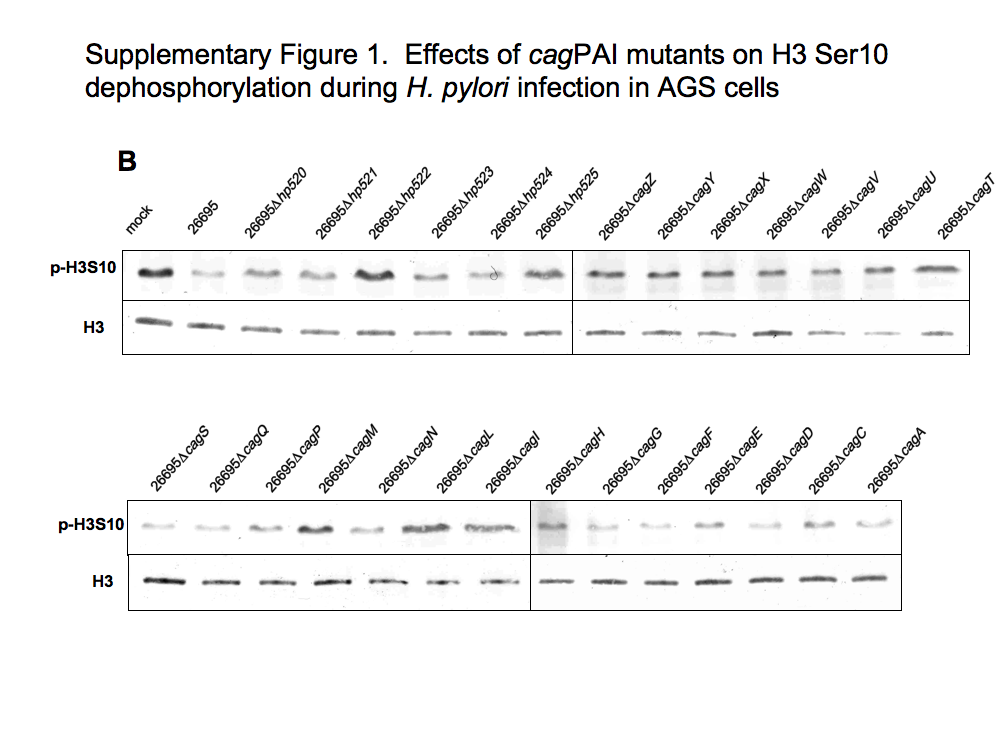

Supplement: Figure S1 — Effects of cagPAI mutants on H3 Ser 10 dephosphorylation during H. pylori infection in AGS cells. AGS cells (5×10^5^) were treated in the presence or absence of wild-type H. pylori 26695 and its various mutants strains at MOI of 150:1. The cell lysate was then subjected to immunoblot analysis with rabbit anti-phospho-histone H3 Ser10 antibodies, anti-total H3 antibodies were used to re-probe the membrane and monitor protein loading. A representative blot from each strain is presented. (0.18 MB TIF) [file pone.0009875.s001.tif]

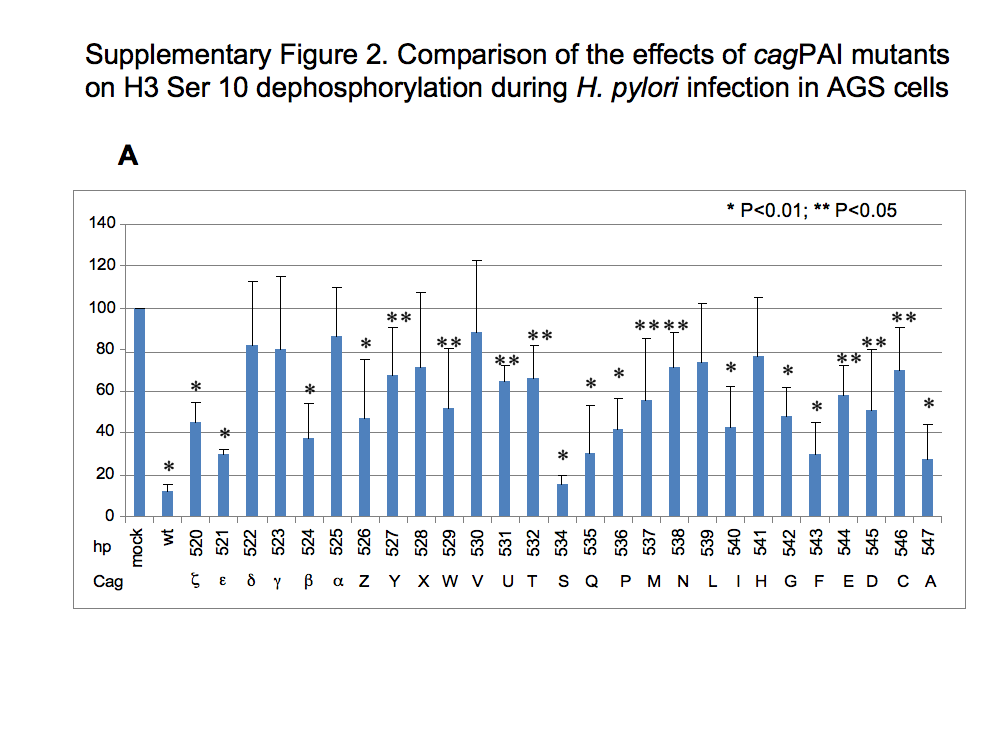

Supplement: Figure S2 — Comparison of the effects of cagPAI mutants on H3 Ser 10 dephosphorylation during H. pylori infection in AGS cells. Data are mean±SEM from 3–6 densitometry scans, adjusted with total histone H3, and expressed as fold changes over the appropriate control. **P<0.05, *P<0.01 when compared with controls. (0.25 MB TIF) [file pone.0009875.s002.tif]

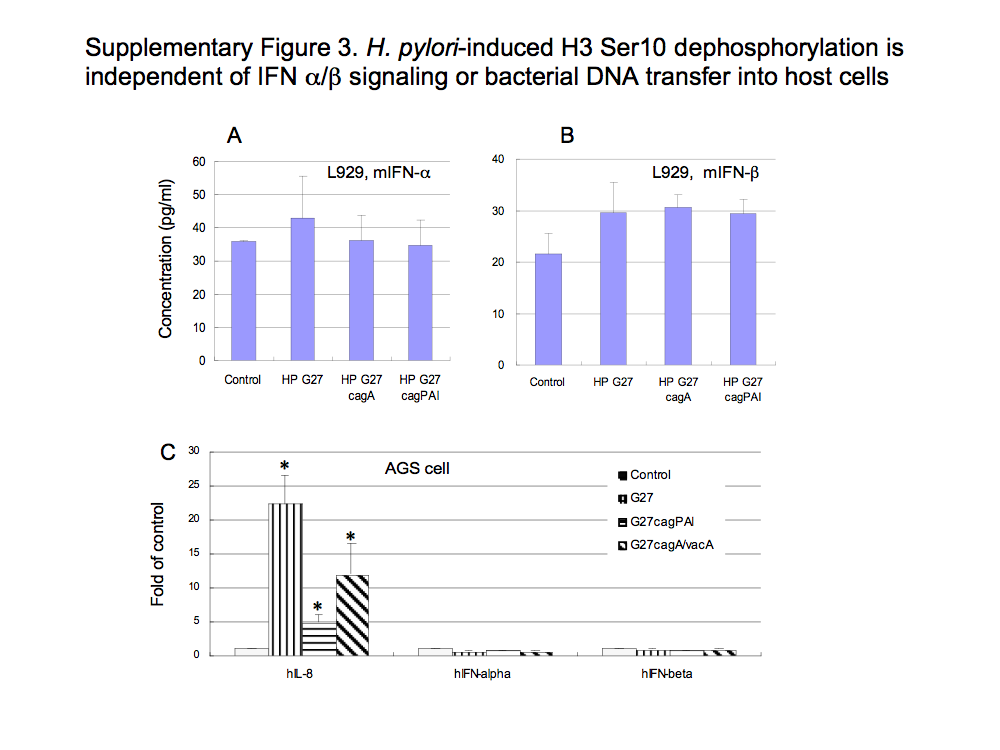

Supplement: Figure S3 — H. pylori-induced H3 Ser10 dephosphorylation is independent of IFN α/β signaling or bacterial DNA transfer into host cells. Mouse fibroblast cell line L929 (2×10^5^) and human gastric epithelial cell line AGS (5×10^5^) were treated with H. pylori G27-MA (G27) or its isogenic mutant strains in antibiotic-free medium for 10 hours at an MOI of 100:1, control cells were treated with medium alone. Supernatant from L929 cells were collected and used to measure IFN α/β production (panels A and B). RNA was extracted from AGS cells with the same treatment and cDNA was made for quantitative RT-PCR assay as described in Materials and Methods. Data are mean±SEM from two duplicate determinants, and PCR data are expressed as fold changes over control without bacteria treatment (panel C). *P<0.01 when compared with controls. (0.21 MB TIF) [file pone.0009875.s003.tif]
